# A facilitation model for implementing quality improvement practices to enhance outpatient substance use disorder treatment outcomes: a stepped-wedge randomized controlled trial study protocol

**DOI:** 10.1186/s13012-020-01076-x

**Published:** 2021-01-07

**Authors:** Megan A. O’Grady, Patricia Lincourt, Belinda Greenfield, Marc W. Manseau, Shazia Hussain, Kamala Greene Genece, Charles J. Neighbors

**Affiliations:** 1 Department of Public Health Sciences, University of Connecticut School of Medicine, 263 Farmington Ave., Farmington, CT 06030-2635 USA; 2 New York State Office of Addiction Services and Supports, 1450 Western Ave., Albany, NY 12203 USA; 3 New York State Office of Addiction Services and Supports, 501 7th Ave., 8th Floor, New York, NY 10018 USA; 4 Partnership to End Addiction, 485 Lexington Avenue, 3rd Floor, New York, NY 10017-6706 USA; 5 Department of Population Health, NYU Grossman School of Medicine, 180 Madison Avenue, New York, NY 10016 USA; 6 NYU Wagner Graduate School of Public Service, 295 Lafayette Street, New York, NY 10012 USA

**Keywords:** External facilitation, Quality metrics, Opioid use disorder, Quality improvement, Implementation, Stepped-wedge trial

## Abstract

**Background:**

The misuse of and addiction to opioids is a national crisis that affects public health as well as social and economic welfare. There is an urgent need for strategies to improve opioid use disorder treatment quality (e.g., 6-month retention). Substance use disorder treatment programs are challenged by limited resources and a workforce that does not have the requisite experience or education in quality improvement methods. The purpose of this study is to test a multicomponent clinic-level intervention designed to aid substance use disorder treatment clinics in implementing quality improvement processes to improve high-priority indicators of treatment quality for opioid use disorder.

**Methods:**

A stepped-wedge randomized controlled trial with 30 outpatient treatment clinics serving approximately 2000 clients with opioid use disorder each year will test whether a clinic-level measurement-driven, quality improvement intervention, called Coaching for Addiction Recovery Enhancement (CARE), improves (a) treatment process quality measures (use of medications for opioid use disorder, in-treatment symptom and therapeutic progress, treatment retention) and (b) recovery outcomes (substance use, health, and healthcare utilization). The CARE intervention will have the following components: (1) staff clinical training and tools, (2) quality improvement and change management training, (3) external facilitation to support implementation and sustainability of quality improvement processes, and (4) an electronic client-reported treatment progress tool to support data-driven decision making and clinic-level quality measurement. The study will utilize multiple sources of data to test study aims, including state administrative data, client-reported survey and treatment progress data, and staff interview and survey data.

**Discussion:**

This study will provide the field with a strong test of a multicomponent intervention to improve providers’ capacity to make systematic changes tied to quality metrics. The study will also result in training and materials that can be shared widely to increase quality improvement implementation and enhance clinical practice in the substance use disorder treatment system.

**Trial registration:**

Trial #NCT04632238NCT04632238 registered at clinicaltrials.gov on 17 November 2020

Contributions to the Literature

- We will advance the literature on the implementation of quality improvement practices by testing whether a multicomponent intervention improves high priority targets for quality measurement in substance use disorder treatment (e.g., 6-month retention in care).
- We will make a novel contribution to the literature by testing whether incorporating a technology-based client-reported treatment progress tool (the Treatment Progress Assessment-8) supports data-driven clinical decision making and clinic-level quality measurement and improvement.
- We will extend the external facilitation literature by adapting the Integrated Promoting Action on Research Implementation in Health Services (i-PARIHS) framework to implement quality improvement practices in substance use disorder treatment programs.

## Background

The misuse of and addiction to opioids is a national crisis that affects public health as well as social and economic welfare. Data from 2018 shows that 128 people in the USA died every day from opioid overdoses and approximately 2 million people had opioid use disorder (OUD) [1]. The opioid epidemic has strained the healthcare and substance use disorder (SUD) treatment systems. For example, from 2010 to 2017, OUD-related hospitalizations increased by 54% and emergency department visits increased by 109% [2]. During this same time, admissions to SUD treatment programs for opioid use grew by 42% [3]. The current COVID-19 pandemic has caused additional concern due to anticipated increases in opioid use and overdose deaths as well as decreases in treatment access [4–10].

Multiple systematic reviews have concluded that SUD treatment in the USA has large gaps in quality of care and limited capacity for systems-level improvement [11–16]. These reviews and other studies highlight a number of systemic issues in the SUD treatment system, including (1) too few with an SUD have access to treatment, (2) treatment is often not evidence based, and (3) poor treatment completion and retention rates among those who do access treatment [17–19]. These quality gaps could be partially due to the SUD treatment workforce traditionally having limited professional education and training to provide scientifically supported practices or engage in quality improvement (QI) initiatives to change clinical outcomes [11, 14, 15]. Further, clients cite lack of flexibility and care that is not person-centered as reasons for dissatisfaction and lack of engagement in treatment [20–23]. Additionally, there have been few financial incentives to drive innovation among SUD treatment programs.

As a result of the issues described above, there have been consistent calls for new payment models that link payments to measures of quality instead of fee-for-service payment models that reward volume [11, 14]. In order to transition to such payment models, programs must incorporate or improve evidenced-based practice offerings, offer more person-centered approaches, become more data and outcome-driven, implement QI programs, and expand the use of technology [13, 24–27]. Yet, there is deep concern that SUD treatment programs cannot survive this transition and may need a great deal of support to improve their clinical and business operations [28, 29]. Therefore, the workforce could benefit from training on evidence-based practices and person-centered care, new tools to better monitor client treatment outcomes, and support to implement QI protocols.

There is limited research on how to best guide SUD treatment programs in implementing systematic QI processes [30, 31]. Challenges to implementing QI protocols in SUD treatment programs include non-prioritization of data collection, limited analytical capacity, poor IT systems, and lack of data-driven decision-making culture [32, 33]. Qualitative research has found that SUD treatment staff need basic training on data collection and use [34]. One of the most studied approaches to QI in SUD treatment, the Network for Improvement of Addiction Treatment (NIATx), emphasizes identifying key problems, engaging change leaders, and using a series of rapid cycle tests to make changes. While the NIATx approach has been shown to improve service provision in its targeted areas (i.e., wait times and retention), there are mixed results for other outcomes (e.g., increasing admissions) [34–36] as well as criticisms, including the level of commitment required by staff, scarce resources to implement burdensome processes, detailed data requirements, and issues with long-term sustainability [30, 34, 37]. New strategies are needed to guide SUD treatment programs through implementing sustainable QI processes that build on lessons learned from previous research and that are tailored to existing resource and workforce limitations.

Utilization of data and metrics as well as implementation of QI processes will require SUD clinics to make significant changes at various levels within their organizations. This study will use an external facilitation model guided by the Integrated Promoting Action on Research Implementation in Health Services (i-PARIHS) framework to support clinic implementation of QI processes [38, 39]. i-PARIHS posits that optimal implementation occurs when practice facilitation promotes the acceptance and use of a new innovation by tailoring it to the recipient’s specific needs [39, 40]. Facilitators are the active ingredient that help navigate teams through complex change processes by addressing (a) the fit within the existing clinic, (b) motivations, beliefs, goals, and resources of intervention recipients, and (c) the inner and outer implementation context. In QI projects, the goal of facilitation is typically to support sustained focus and achievement of specific goals; facilitators work to enable individuals and teams to analyze, reflect, and change their way of working [41].

Patient monitoring tools are central to managing quality of care for other behavioral health conditions yet are not often systematically used in SUD treatment programs. For example, a hallmark of collaborative care models for depression is the use of evidence-based assessment tools, like the PHQ-9 depression scale, for ongoing monitoring and treatment adjustments. We recently developed an 8-item tool for SUD clinic settings, the Treatment Progress Assessment-8 (TPA8), that monitors SUD symptoms and treatment progress indicators that are associated with clinical outcomes and relapse [42]. We have been in the process of developing an electronic version, the eTPA8, to ease administration to clients as well as to provide a robust data reporting system that SUD clinics can use in client care as well as QI efforts. The eTPA8 will be part of the intervention package in the current study to support QI practices.

**Fig. 1:**
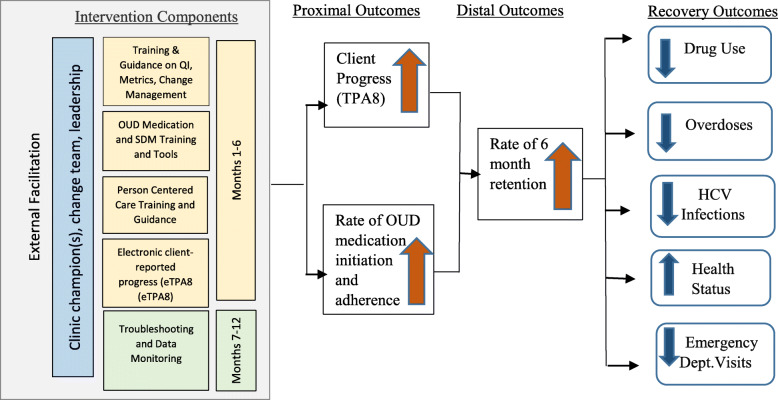
Intervention conceptual method

In this paper, we describe a study protocol for a stepped-wedge randomized controlled trial (SW-RCT) stemming from a state-academic partnership that will test a multicomponent, clinic-level intervention, called Coaching for Addiction Recovery Enhancement (CARE). We will test whether CARE improves treatment process quality and recovery outcomes over treatment as usual, particularly for OUD. The SW-RCT design was selected mainly because the intervention is conducted at the clinic-level and, from a resource perspective, it would be impractical to provide the intervention to all clinics at once given the facilitation model being utilized. Further, the stepped-wedge design was seen as more acceptable and ethical for key program stakeholders (notably the single state agency that regulates SUD treatment [SSA]) due to the potential benefits from the resources being provided as part of the intervention package (e.g., training support from the SSA) compared to a parallel randomization design. The CARE intervention includes the following components: (1) staff clinical training and tools, (2) quality improvement and change management training, (3) external facilitation to support implementation and sustainability of quality improvement, and (4) the eTPA8 client-reported treatment progress tool to support data-driven decision making and clinic-level quality measurement. See Fig. 1 for our intervention conceptual model. We describe intervention components in more detail in the method section. Study aims and hypotheses are the following:

- Aim 1: Test the effect of CARE on treatment process quality measures and recovery outcomes.
  - Hypothesis 1: CARE will improve clinic-level rates of treatment process quality measures (i.e., initiation of and adherence to OUD medications, in-treatment symptom and therapeutic progress, 6-month treatment retention) and recovery outcomes (i.e., substance use, overdoses, self-reported health status, hepatitis C infection [HCV], and emergency department [ED] visits).
- Aim 2: Examine association of changes in the eTPA8 client-reported treatment progress tool with treatment retention and substance use outcomes.
  - Hypothesis 2: Positive change in the eTPA8 scale will be associated with longer retention and reduced substance use at follow-up.
- Aim 3: Examine whether there are program admission changes in terms of client complexity (e.g., homelessness, psychiatric conditions) to test impact on client access (i.e., adverse selection) as clinics monitor quality measures.
  - Hypothesis 3: Clinic rates of severe OUD (i.e., daily use and/or injection use), homelessness and psychiatric conditions at admission during the year following the CARE intervention will decrease from baseline rates.
- Aim 4: Examine staff attitudes, experiences, and behaviors related to (1) implementing the eTPA8 and clinical practice change, and (2) working with external facilitators to implement QI processes.
- Aim 5: Estimate addiction program and state intervention costs of CARE and impact on Medicaid costs.

## Method/design

### Design and participants

**Fig. 2:**
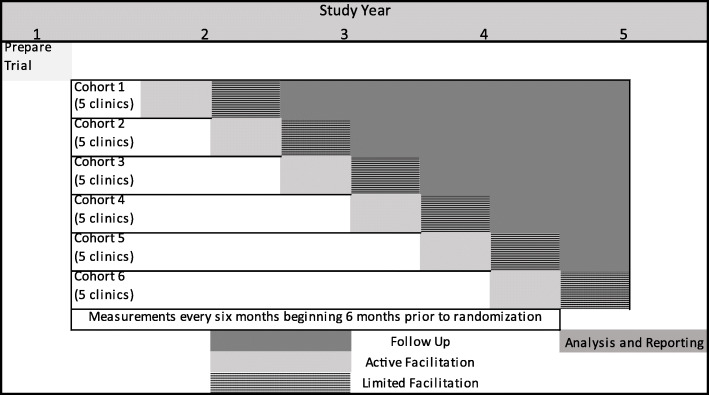
Stepped-wedge design

We will employ a SW-RCT with 30 outpatient SUD treatment clinics who serve approximately 2000 clients with OUD per year to test whether CARE improves treatment process quality and recovery outcomes over treatment as usual. The study will benefit from multiple sources of data to test study aims, including state administrative data, client-reported survey and eTPA8 data, and staff interview and survey data. Therefore, this will be a robust study of the implementation of a measures-driven QI approach for SUD treatment. Figure 2 shows the stepped-wedge design. Five clinics will start CARE every 6 months over 3 years; therefore, there are 6 steps, each with 5 clinics randomly assigned to 1 of the 6 steps.

Clinics are being recruited from various regions in New York State—Long Island, New York City and its suburbs, the Hudson Valley, and central New York—that have diversity in population density, resources, and racial/ethnic representation. Inclusion criteria will be outpatient treatment programs licensed by the NYS Office of Addiction Services and Supports (NYS OASAS) with a minimum of 50 OUD clients per year. For-profit clinics will be excluded because of insufficient numbers within New York and differences in organizational culture. To recruit clinics, we first identified 77 eligible clinics based on our inclusion criteria using state administrative data. The study PIs and project director then invited these identified clinics via email to informational webinars about the study, emailed them information sheets describing the study, and reached out via personal emails to clinic leaders. NYS OASAS representatives from the study regions also sent informational emails to eligible clinics. To finalize recruitment, clinics who agree to participate will sign a clinic participation agreement form. Clinic randomization will be stratified by downstate (Long Island, New York City, and its suburbs) and rest-of-state (Hudson Valley and central New York). We will use the SAS PROC PLAN procedure to generate the randomization schedule; a data analyst at NYS OASAS not associated with the study will conduct this procedure to minimize bias.

### Intervention planning and pilot

This study was funded under a phased NIH award such that there was a 1-year developmental phase that was recently successfully completed, allowing for a second phase of funding for the SW-RCT described in this paper. During this 1-year developmental phase in 2019–2020, we conducted two stakeholder meetings, piloted CARE study components, tested and finalized the eTPA8 client-reported treatment progress tool, and created study materials and protocols. The stakeholder meetings were conducted to get input and feedback about the CARE intervention and included attendees from New York City, State and County agencies, clients with lived experience in SUD treatment, insurers, academic partners, and SUD treatment providers. A 3-month pilot was conducted in an outpatient clinic during which we tested components of the CARE intervention for feasibility, including engaging executive leadership, providing clinical and QI trainings, and using an external facilitator to engage clinic champions and a change team in a QI cycle. Focus groups were conducted to understand staff experiences during the CARE pilot, and intervention materials and protocols were finalized. Finally, we tested and finalized the eTPA8 client outcome monitoring tool by using cognitive testing procedures with pilot clinic clients and testing the tool on a web-based usability program [43–45]. We also asked pilot clinic staff for feedback on the eTPA8.

### Description of the CARE intervention

The overall goal of the multicomponent, clinic-level CARE intervention is to help SUD clinics make improvements in rates of OUD medication initiation and adherence, rates of 6-month retention, and recovery outcomes by providing training and implementing QI procedures with support from an external facilitator. The CARE intervention will be 1-year-long for each participating clinic. Guided by phased implementation models [46], ‘active’ intervention by the external facilitator will take place during the first 6 months (see Table 1). The second 6 months will be a sustainment phase in which clinics continue QI activities with limited facilitator support (e.g., troubleshooting). Guided by the i-PARIHS model, facilitators will support clinics in making adjustments to clinical and program procedures. They will tailor their approach to fit with each clinic’s workflow while addressing staff motivations and resources and attending to the inner and outer context of each clinic.

**Table 1:** CARE intervention components during 6-month active phase

CARE component	Rationale	Execution	Target
Training/tools on OUD medications, shared-decision making, and person-centered care	Increase knowledge, skills, and, attitudes about evidenced-based practices to improve client outcomes	1-h, interactive web-based trainings; moderated discussion with external facilitator; guides/tools for OUD medication and shared decision making	All clinic staff
Leadership engagement	Gain buy-in from executive level of the organization to ensure resources available for QI processes and clinical practice change	1.5-h kick-off meeting with study team and ongoing updates and engagement from external facilitator and champions	Clinic executive leadership
Establish clinic champion(s) and change team	Structured team to conduct QI and change management activities	Executive and clinic leadership select champion(s) with support from facilitator	Identified clinic staff
Training on QI and change management	Improve numeracy, data management, and quality improvement knowledge	45-min web-based training plus external facilitator provides training to champion; champion provide training to change team	Clinic champion(s) and change team
Ongoing QI and change management activities	Improve clinics’ ability to engage in data-driven, structured QI cycles and ongoing data monitoring	Weekly 1-h change team meetings lead by clinic champions and supported by the external facilitator	Clinic champion(s) and change team
eTPA8 client-reported treatment progress tool	Improve clinical outcomes and capacity for technology-based data monitoring and QI	Clients complete the eTPA8 every 2 weeks; staff monitor reports for use in clinical decision making; change team uses reports for QI	All clinic staff, champions, change team

### Measures and materials

#### Data sources

The NYS OASAS registry of all admissions and discharges (irrespective of payer) to licensed addiction treatment facilities—the Client Data System (CDS)—forms the basis of our administrative data set. The CDS will record all treatment episodes that occur in the 30 clinics we include in this study, providing a rich source of data. All licensed providers of SUD treatment in NYS are mandated to submit admission and discharge data into the CDS. NYS OASAS tracks each individual by unique identifiers. When clients enter treatment, providers enter information on demographics, level of functioning (e.g., health status, comorbidities), criminal justice status, recent history and frequency of substance use, and recent SUD treatment history. Upon discharge, providers enter information on functional status (e.g., employment, housing), source of payment for treatment (e.g., self-pay, Medicaid, private insurance), criminal justice involvement during treatment, hospitalizations, and use of ED services. Additionally, the discharge information has type, frequency (i.e., number of sessions), and intensity of SUD treatment as well as discharge status (discharge disposition and goal achievement). NYS Medicaid claims will supplement the administrative dataset by providing data on OUD medications and health services use (e.g., ED visits). We will link clients in Medicaid to the CDS using the NYS OASAS identifier augmented, in cases where there were no exact matches, by searching for same treatment events that were recorded in each database. Additionally, we will employ probabilistic matching techniques to account for data entry errors on elements of the OASAS identifier. Our link rate in previous studies among OUD individuals in Medicaid is 90% [47].

Two additional data sources will represent client-reported outcomes. First, the 8-item eTPA8 will be completed by clients in participating clinics every 2 weeks; data will be retained in the eTPA8 database for analysis. Second, a sample of 40 clients from each clinic will be recruited to complete a survey: 20 clients at the beginning of the intervention and another 20 6 months after the intervention start date at their clinic. Each participant will be assessed three times: (1) at treatment entry, (2) 3 months post-entry, and (3) 6 months post-entry. We will use a mobile platform to collect this data. To recruit clients, clinic staff will present a recruitment flyer to randomly selected newly admitted clients with a primary or secondary OUD diagnosis. The flyer will describe the study and provide a number where they can text to participate. Participants will digitally consent and complete the baseline assessment on their mobile phone. If they do not complete the assessment, they will be sent reminder text messages at 3 and 7 days. Clients will receive a text message with a link to the assessment at each follow-up point, as well as text reminders if they do not complete the follow-ups. Clients will be compensated with a $20 gift card for each assessment.

We will examine staff attitudes, experiences, and behaviors related to implementing CARE with the external facilitator. There will be three data sources: (1) surveys, (2) semi-structured interviews, and (3) external facilitator notes, surveys, and checklists [48]. Online surveys of clinic staff will be administered at baseline and 6 and 12 months post-baseline. All part- and full-time staff will be eligible. Semi-structured interviews will be conducted via phone with at least two clinical and one administrative staff (i.e., clinic director or clinical supervisor) at each of the 30 participating clinics approximately 6 months after starting the CARE intervention or until saturation is reached (no new themes or insights occurring). We will use purposeful, criteria sampling for the interviews to select staff who participated in the change team and/or implementation activities as part of CARE. We will follow best practices for phone-based qualitative interviews in health services [49, 50]. Staff members will provide verbal consent for interviews and digital consent for surveys. In order to inform replication and dissemination of this intervention model as well as to inform on CARE experiences, the external facilitator will complete surveys after each site visit/interaction, monthly narrative reports, and tracking of clinic participation in activities modeled on other facilitation studies [48].

#### Measures

Based on treatment episode and client admission information in the CDS and claims information in Medicaid, clinic level covariates, quality, and outcomes data will be computed in 6-month assessment windows. Client level covariates from the CDS will include frequency of use, injection drug use, age, gender, race/ethnicity, education, residential stability/homelessness, and psychiatric comorbidities at admission. Medicaid claims will be used to identify OUD medications, various health conditions based on ICD-10 diagnosis fields; healthcare episodes based on procedure, rate and NDC codes; and cost based on Medicaid payments for claims and encounters. Clinic rates of 6-month retention will be computed from admission and discharge dates in the CDS. OUD medication initiation and adherence will use data from Medicaid pharmacy claims during an episode. Initiation will be a new OUD medication pharmacy claim within 30 days and adherence will be computed using the NQF 3175 definition [51–53]. Overdoses will be identified by CDC defined combinations of diagnoses and acute care procedure codes [54, 55]. All-cause ED visits will be defined by procedure and rate codes. HCV infections will be defined by diagnoses during any hospitalizations or on two outpatient claims [56, 57]. Costs of healthcare services will be drawn from Medicaid claims and encounter data.

Provider-level covariates will also be calculated. SUD program characteristics will be drawn from computing provider-level statistics from claims as well as from state databases that contain addresses, affiliations, and licensing information. Provider variables will include geographical location, annual census, and hospital affiliation. Geographical information of provider location will be drawn from the Area Health Resources File: poverty, population density, ethnic/racial population, and health professional availability.

Client-reported treatment process improvement will be computed from change scores on the eTPA8 from baseline to latest administration. Further, the client-self reported mobile survey will include the following measures: (1) past 2-week use of opioids and other substances based on the Addiction Severity Index (ASI) [58], (2) substance use consequences based on the Short Inventory of Problems-Revised (SIP-R) [59], (3) health-related quality of life based on the first item of the SF-36 [60], (4) functional outcomes using the Treatment Effectiveness Assessment [61], past 30-day employment, incarceration, housing status based on the ASI, (5) a visual analog scale measuring cravings to use substances [62], and (6) demographics.

Staff surveys will include measures on practice change capacity (i.e., the change process capability questionnaire) [63, 64], the Opinions About MAT Survey [65], knowledge and perceptions about person-centered care and shared-decision making, and use of data and quality metrics. An interview guide for staff qualitative interviews will be created to reflect the dimensions of the i-PARIHS model (e.g., inner context) [66].

The marginal cost of the CARE intervention will be estimated to inform future intervention development and implementation. We will collect detailed data on staffing and other resources to inform micro-costing computations and estimates of the marginal costs of deploying CARE under real-world conditions [67–69]. Cost data collection will follow generally accepted practice in cost-effectiveness research [67, 68]. In addition, the cost collection procedures and estimations will borrow from methods derived from process engineering [70] and managerial accounting [71–74]. Input prices (i.e., cost of each unit of resource input) will be derived from market rates when available, program budgets, or government pricing schedules (e.g., Bureau of Labor Statistics data on personnel costs for the Northeast region). The first step in the cost tracking will entail developing detailed process maps in conjunction with intervention developers and clinicians to identify all key processes [75]. The cost tracking system will track resources used to deliver the intervention as well as identify research protocol driven costs that would not be part of a real-world implementation [76]. The cost tracking system will identify intervention development costs as well as operational costs associated with running the intervention.

### Analysis and power

The primary aim of this study is to test whether CARE is associated with greater use of pharmacotherapy for OUD, longer retention in care, and improved recovery outcomes (e.g., reduced drug use). We will employ multiple convergent analytical methods to (a) examine CARE implementation and (b) test association between CARE and OUD treatment outcomes. Analyses will begin with examination of distributions of variables and transformations where appropriate, examination of patterns of missingness, and application of formal imputation methods [77–79] if deemed essential to project aims. Analyses for Aims 1, 2, and 3 will utilize general linear mixed models (GLMM) for stepped-wedge designs [80–85]. For example, to test the effect of CARE on treatment process quality measures (i.e., initiation and adherence to OUD medications, in-treatment symptom and therapeutic progress, 6-month treatment retention) and recovery outcomes (substance use, overdoses, self-reported health status, HCV infection, and ED visits), we will employ stacked difference-in-difference models that test for within and across clinic changes while adjusting for secular trends across time [80, 86, 87]. The models will take the form: *Y*_*ikt*_ = *μ* + *X*_*kt*_*θ* + *β*_*t*_ + *Z*_*ikt*_ + *α*_*k*_ + *e*_*ikt*_, where *β*_*t*_ is a fixed effect for time, *Z*_*ijk*_ is a matrix of covariates measuring individual, geographical, and clinic characteristics for person *i* in clinic *k* at time *t, X*_*kt*_ is an indicator for intervention in clinic *k* at time *t* (coded 0 for baseline then 1 from when intervention begins in clinic through end of study), and *θ* is the treatment effect. *Y*_ikt_ represents a matrix of binary variables: treatment process quality measures (i.e., pharmacotherapy initiation and adherence, in-treatment symptom and therapeutic progress, treatment retention) and recovery outcomes (i.e., substance use, overdoses, self-reported status, HCV infection, and ED visits). We will estimate the effect of CARE (*θ*) while controlling for secular trends (*β*) and adjusting for clustering within clinic (*α*_*k*_) using robust standard errors. We will also conduct a set of falsification tests. These will include (1) testing for variation in program and client characteristics across time of enrollment; (2) examination of variation across study time in the study outcomes for clinics not participating in the study; and (3) examination of variation in intervention effect based on clinics’ baseline levels of quality measures (e.g., comparing top third to bottom third in quality of care at baseline).

To examine Aim 4, we will use a concurrent triangulation mixed-methods design such that staff quantitative and qualitative data collection will occur concurrently and results will be integrated after analysis of each [88]. The semi-structured interviews will be transcribed and analyzed using conventional qualitative content analysis [89] following the process outlined in Erlingsson and Brysiewicz [90]. Atlas.ti will be used to manage the data. A codebook will initially be created based on i-PARIHS constructs and will be modified and amended as coding and analysis continues. For the quantitative results, multilevel modeling will be used to examine change over time in survey scores given the nested structure of the data (staff nested within clinics) [91].

For Aim 5, the cost analysis will focus on the provider and state agency perspective as the main decision-maker; consequently, other societal costs are not included in this preliminary analysis. Decision analytical models will be created using measures of central tendency (e.g., means, medians) of observed resource inputs as well as measured estimates of variances for sensitivity analyses. Using TreeAge Pro [92], we will estimate average costs as well as confidence intervals based on simulations drawing from variances of model inputs. We will conduct a series of one-way and two-way sensitivity analyses across model inputs to determine the impact of variability of individual model parameters. Additionally, we will use Monte Carlo simulation to build confidence intervals over which the cost of the intervention may vary. Costs will be presented as ratios: per clinic, per participant, and per unit of each quality measure.

We will also examine the association between CARE and total Medicaid costs. Costs will be modeled using generalized gamma models (GGM), which offer flexibility in modeling non-Gaussian outcomes by computing three parameters to fit the observed distribution: location, scale, and shape [93, 94]. One advantage of GGM is that it is not subject to the retransformation bias stemming from heteroscedasticity in the log-scale often associated with the more common OLS regression on log-transformed healthcare costs [94–96]. We will consider other GLM approaches to analyzing costs and subject these to goodness of fit tests as outlined by Manning [97].

To estimate study power for select outcomes, we drew from methods developed by Hussey and Hughes [80] as implemented in a Stata command, stepped wedge [98]. Using administrative data to estimate sample sizes and baseline rates (μ), we computed detectable differences for a β = 0.80, α = 0.01, ICC/ρ = 0.20, and SW-RCT with 6 crossover points, and 5 clinics randomized at each point. For 6-month retention, assuming step samples sizes (*m*_k_) of 132 and base rate (μ) of 25%, we will be able to find a 2.8pp change in retention (i.e., 27.8%) to be statistically significant and clinically meaningful. We also conducted sensitivity analyses varying ρ (0.10–0.30) and β (0.90) and found detectable differences ranging from 2.6 to 3.2%. For OUD medication initiation, we assume *m*_k_ = 40 Medicaid clients with OUD, μ = 0.40, and find a detectable difference of 6.7%. For ED visits, we assume *m*_k_ = 79 Medicaid clients and μ = 0.40, and find a detectable difference of 3.9%. Consequently, with 30 clinics and 6 steps in our SW-RCT, we will have power to detect moderate and clinically meaningful differences in outcomes.

## Discussion

This study will provide a robust examination of the implementation of a measures-driven QI approach for SUD treatment. We will be able to provide insight into whether a multicomponent intervention that includes clinical training, training and support on QI processes, external facilitation, and an electronic client outcomes monitoring system improves quality and recovery outcomes. The study benefits from a strong academic-state partnership, an external facilitation model guided by the i-PARIHS implementation science framework, multiple sources of data to examine impacts of the intervention, as well as tools and trainings developed with input from a variety of stakeholders and a pilot during the first phase of this project.

The study team is currently finalizing clinic recruitment and randomization with plans to start the first five clinics in the intervention in December 2020 and January 2021. The ongoing COVID-19 pandemic has forced a shift in SUD treatment service delivery, which has traditionally relied heavily upon in-person services. Outpatient treatment clinics have had to rapidly and fundamentally change their service delivery models due to physical distancing mandates and new COVID-19 safety protocols. For example, programs have needed to change their work flows to accommodate telepractice for induction and management of medications for OUD, provide individual and group counseling via telemedicine, reduce reliance on drug screening (i.e., toxicology testing), and employ new strategies for treatment engagement. Therefore, while the pandemic continues, this study will not only have to adjust some of its facilitation and training activities to be done remotely rather than in-person, but we will also tailor facilitation to assist clinics in improving quality of care in the context of COVID-19 shifts in service delivery and workflows. We will carefully document changes to our protocols and clinic procedures as the COVID-19 pandemic continues to affect NYS and our participating project sites.

While there are a number of strengths in this study, we acknowledge the following possible exogenous and endogenous threats to validity. First, administrative data is incomplete. We rely significantly on administrative data to increase the efficiency of our research study that tests the impact of CARE in 30 clinics on treatment quality measures (e.g., pharmacotherapy use, retention) and outcomes (e.g., relapse). While there is strong precedent for using administrative data for research [99–106], these data will not capture all aspects of client experience (e.g., substance use, social consequences). To address the limitation, we are using a mobile platform that allows for collection of research data from a subsample of participants. These data will allow us to further study the intervention impact on recovery outcomes not captured in the administrative data.

In addition, there may be variability in the effect of the intervention due to variation in individual clinic capacity to integrate QI practices within their organizational structure. It will be critically important for future dissemination efforts to understand factors implicated in the implementation of CARE. While the study has adequate statistical power to detect a main effect, it will be important to understand the variability in effect across clinics. To address this, we will incorporate the embedded mixed-methods component of the study examining staff experiences. Additionally, we will explore potential intervention-related factors by conducting a series of post hoc analyses.

Finally, though the SW-RCT design offers a number of benefits, clinics will be randomized to step at the start of the trial with start dates staggered across 3 years. Therefore, it may be challenging to retain recruited clinics in the study while they wait to start the intervention. To reduce clinic attrition, we will employ strategies recommended in the stepped-wedge trial literature (e.g., keep recruited sites informed of progress, hold regular pre-intervention meetings) [107]. Also, recruited clinics will sign a study agreement indicating they understand the randomization procedures and timeline.

In summary, SUD clinics are under pressure to transition into a system that values quality metrics and outcomes. However, the workforce has been under-resourced and undertrained in processes that could assist them in making systemic changes to improve clinic functioning and client outcomes. This study will provide the field with a strong test of a multicomponent intervention to improve providers’ capacity to make systematic changes tied to quality metrics. The study will also result in training and materials that can be shared widely to improve QI implementation and clinical practice in the SUD treatment system.

## Data Availability

The full protocol can be accessed by contacting the corresponding author.

## Abbreviations

CARE: Coaching for Addiction Recovery Enhancement
ED: Emergency department
GGM: Generalized gamma models
GLMM: General linear mixed models
HCV: Hepatitis C virus infection
i-PARIHS: Integrated Promoting Action on Research Implementation in Health Services
NIATx: Network for Improvement of Addiction Treatment
OASAS: New York State Office of Addiction Services and Supports
OUD: Opioid use disorder
QI: Quality improvement
SUD: Substance use disorder
SW-RCT: Stepped-wedge randomized controlled trial
TPA8: Treatment Progress Assessment-8
